# Decoding the Heart Through Computed Tomography: Early Cardiomyopathy Detection Using Ensemble-Based Segmentation and Radiomics

**DOI:** 10.3390/jimaging12030120

**Published:** 2026-03-10

**Authors:** Theodoros Tsampras, Alexios Antonopoulos, Theodora Karamanidou, Georgios Kalykakis, Konstantinos Tsioufis, Charalambos Vlachopoulos

**Affiliations:** 1 Cardiogenetics and Sports Cardiology Unit, 1st Cardiology Department, Hippokration Hospital, National and Kapodistrian University of Athens, 11527 Athens, Greece; teodore.tsampras@gmail.com (T.T.); antonopoulosal@yahoo.gr (A.A.);; 2 Pfizer Center for Digital Innovation, 55535 Thessaloniki, Greece

**Keywords:** deep learning, myocardial segmentation, radiomics, cardiomyopathies

## Abstract

Diagnosis of cardiomyopathies often depends on overt phenotypic manifestations, delaying patient management. This underscores the need for population-level opportunistic screening tools using clinically indicated CT scans to detect subclinical myocardial disease. This study developed an Ensemble Machine Learning (ML) model to automatically segment the left ventricular myocardium from CT data and estimate the probability of underlying myocardial disease using radiomic feature analysis. A total of 60 CT scans (~12,000 images) were used to train ML models for left ventricular myocardium segmentation, including scans from both healthy individuals and patients with myocardial disease. A novel Ensemble model was developed and externally validated on 10 independent CT scans. Subsequently, 100 unseen CT scans were segmented manually and automatically for radiomic feature analysis. After removing highly correlated features through intra-class variation and correlation filtering, the refined dataset was used for model training and testing. Key predictive features were identified, and model performance was evaluated. The four best-performing models (Unet++, ED w/ASC, FPN, and TresUNET) were combined to form an Ensemble model, achieving a final DICE score of 0.882 after hyperparameter optimization. External validation yielded a DICE score of 0.907. Radiomic feature analysis identified 15 key predictors of myocardial disease in both manual and automatic segmentation datasets. The model demonstrated strong performance in detecting underlying myocardial disease, with AUCs of 0.85 and 0.8, respectively. This study presents a fully automated CT-based framework for LV myocardial segmentation and radiomic phenotyping that accurately estimates the probability of underlying myocardial disease. The model demonstrates strong generalizability across different CT protocols and highlights the potential role of AI-driven CT analysis for early, non-invasive cardiomyopathy screening at a population level.

## 1. Introduction

Cardiomyopathies are a diverse group of myocardial diseases, now recognized as significant contributors to heart failure [1,2]. However, clinical diagnosis is typically delayed, primarily because it depends on the development of phenotypic traits of the disease [3,4,5,6]. This delay underscores the need for screening tools that can identify subclinical myocardial diseases before overt symptoms emerge. In this context, machine learning (ML) and radiomic analysis have gained traction as they show potential for early detection and disease classification [7].

Recent advances have explored the use of ML algorithms for automatic segmentation of the left ventricular myocardium across various imaging modalities. While Cardiovascular Magnetic Resonance (CMR) is a valuable tool for cardiomyopathy screening, and automatic segmentation using CMR scans has been successfully achieved, Computed Tomography (CT) scans offer wider availability, enabling opportunistic screening from routinely acquired, clinically indicated scans [8,9,10,11]. Left ventricular (LV) segmentation allows for the prompt extraction of radiomic features, quantitative data derived from imaging that capture tissue shape and texture characteristics invisible to the naked eye [12]. Through radiomic feature analysis, disease diagnosis and phenotyping can be performed, aiding in non-invasive diagnosis as well as disease monitoring [7].

This study presents an ensemble ML model comprising four individual segmentation models, capable of automatically segmenting the LV myocardium from CT images. Radiomic features extracted from the segmented images are then used to estimate the probability of underlying cardiomyopathy. This study paves the way for earlier intervention and improved patient outcomes in the management of cardiomyopathies.

## 2. Materials and Methods

### 2.1. Data Collection

The dataset for the creation of the automatic LV segmentation algorithm comprised 60 CT scans collected both from individuals without known myocardial disease and from patients with underlying cardiomyopathies, including hypertrophic cardiomyopathy, transthyretin amyloid cardiomyopathy (ATTR-CM), and severe aortic stenosis. This cohort provided a diverse sample for training of the ML model, aiming to increase robustness and generalizability. The CT scans spanned various imaging protocols, including coronary CT angiography, thoracic CT angiography, and thoracic-abdominal CT angiography, to ensure generalization across different imaging protocols.

The dataset used for radiomic feature extraction and myocardial disease classification consisted of 100 CT scans. Among these 100 scans, 50 scans belonged to individuals lacking a documented myocardial disease, while another 50 belonged to patients that were diagnosed with various types of myocardial disease (e.g., hypertrophic cardiomyopathy, ATTR-CM). Manual LV myocardial segmentations as well as automatic LV myocardial segmentations, all derived from the same set of 100 CT scans, were performed. To ensure consistency in the radiomic extraction and analysis, an automatic script for radiomic feature extraction was developed using Python (Version 3.12.3) and the PyRadiomics extension. This script applied identical settings for both manual and automatic annotations, ensuring uniformity in the extraction process. The resulting radiomic feature datasets were used for distinguishing between healthy and diseased myocardial tissue.

### 2.2. Machine Learning Model Development

For the development of an ML model designed to automatically segment the LV myocardium from CT images, a dataset of 60 CT scans (~12,000 images of the heart) was used for model training. Manual segmentations of the myocardium were performed using 3D Slicer software (version 5.6.2) to generate accurate ground truth data. This dataset served as the foundation for training the ML model. A patient-level 10-fold cross-validation scheme was employed on the 60 CT scans to benchmark model performance and ensure robustness across subjects.

To address challenges such as multi-zoom images, image artifacts, and rotations, moderate-to-heavy image augmentation techniques were integrated into the training process. These augmentations included introducing blur, adding noise, applying perspective changes, and warping. Additionally, cropping and zooming were applied to help mitigate the multi-zoom nature of the dataset (due to the inclusion of CT scans with different protocols), ensuring that the model could handle the variability in image quality and perspective. The model’s performance was validated against the manual segmentation dataset. 

After training the model, external validation was performed using 10 new CT scans that were not part of the original training dataset. Manual segmentations of these scans were compared with the automated segmentations produced by the ML model.

### 2.3. Radiomic Feature Extraction and Selection

Radiomic feature extraction was performed in both the manual and the automatic LV segmentation dataset that were generated from the same 100 CT scans. Initially, 843 radiomic features were extracted from each scan. These features included key texture, shape, and intensity features. To ensure the use of the most reliable and consistent features, a statistical evaluation was performed. The process involved intra-class cross-variation analysis, which led to the identification of radiomic features that demonstrated stability across both the manual and automatic segmentations. As a result of this analysis, features with significant cross-variation (>25%) between the two segmentation methods were eliminated. This step was crucial in maintaining the robustness of the radiomic features for predicting myocardial pathology independently of the segmentation dataset used.

A correlation analysis was carried out to address the issue of redundant features, as highly correlated features can lead to model overfitting and reduced generalization performance. Features with a correlation coefficient above 90% were systematically removed. These features represented the most independent and significant predictors of myocardial pathology. The refined feature dataset was used to build a predictive model.

### 2.4. Classification of Myocardial Disease

The refined radiomic feature dataset created was utilized to predict the presence of myocardial pathology. The final dataset, comprising 100 patients and 139 radiomic features per patient, was randomly divided into training and testing sets using an 80/20 split, ensuring that the distribution of normal and abnormal cases remained consistent through stratification. To classify the patients based on the extracted features, a random forest classifier was employed to statistically test the predictive power of the extracted radiomic features. Feature importance was assessed, and 15 key radiomic features were identified as highly predictive of myocardial disease. The model’s performance was evaluated using the Area Under the Curve (AUC) of the Receiver Operating Characteristic (ROC) curve.

## 3. Results

### 3.1. Model Performance Metrics

**Figure 1:**
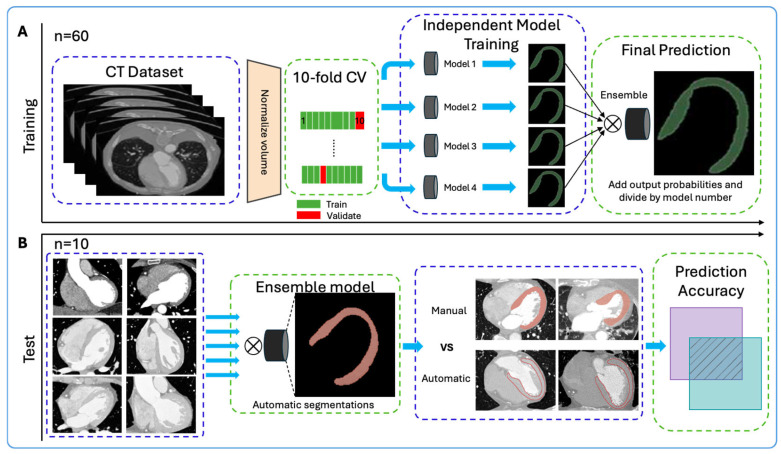
Illustration of the architecture of the Ensemble model for automatic LV segmentation: model training (**A**) and testing process (**B**).

Several machine learning models were evaluated to determine the top performers. Four models, Unet++, ED w/ASC, FPN, and TresUNET, were identified with superior performance metrics. The four independent models showed robust and consistent performance, achieving individual DICE scores of 0.83 to 0.845. These models were subsequently integrated into a novel ensemble, designed to enhance prediction accuracy (Figure 1).

**Figure 2:**
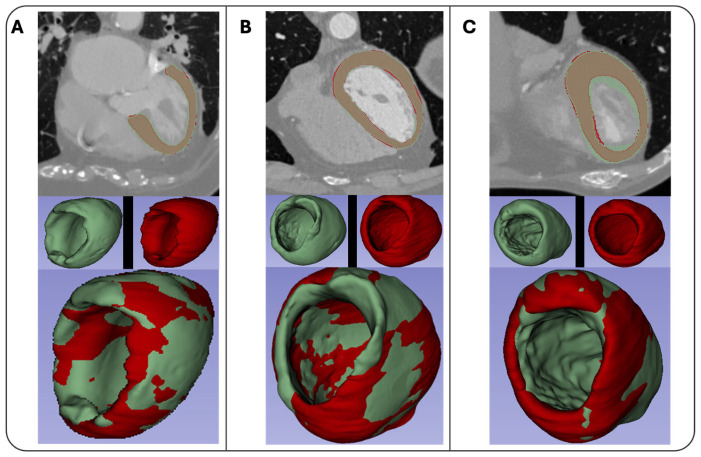
Three segmentation examples (**A**–**C**). The green mask represents the manual annotation, the red mask the segmentation produced by the ML model. The overlap of the two annotations is presented in the mixed-color model.

The various CT protocols introduced different challenges such as variability in zoom levels, contrast, and the presence of artifacts. By incorporating a diverse range of CT scans (CCTAs, ECG-gated CTAs of the thorax, ECG-gated CTAs of the thorax-abdomen) into the training process, the model became more adaptable to handling different CT protocol scans. The Ensemble model achieved a DICE score of 0.882 after completion of model training (Table S1). Examples of the automated segmentations produced by the Ensemble model are illustrated in Figure 2.

Subsequently, an external validation was performed using 10 previously unseen CT scans, which was intended as an initial proof-of-concept to assess the robustness of the ensemble model across heterogeneous CT protocols, scanners, and image characteristics. Given the high slice count per examination, this external validation corresponded to ~2000 cardiac images evaluated at the image level. The model demonstrated strong performance, achieving an average Dice score of 0.907 (Table S2). The inclusion of scans from multiple CT protocols allowed the model to generalize effectively, handling diverse cases that featured differences in zoom levels, artifacts (e.g., Implantable Cardioverter-Defibrillator/Permanent Pacemaker cables), and myocardial tissue pathologies (e.g., hypertrophic cardiomyopathy, ATTR-CM). This broad range of data contributed significantly to the model’s ability to perform consistently across varied imaging environments. The successful external validation confirmed the model’s robustness and was an indication of the model’s capacity to handle real-world CT scans.

### 3.2. Radiomic Feature Extraction and Disease Classification

**Figure 3:**
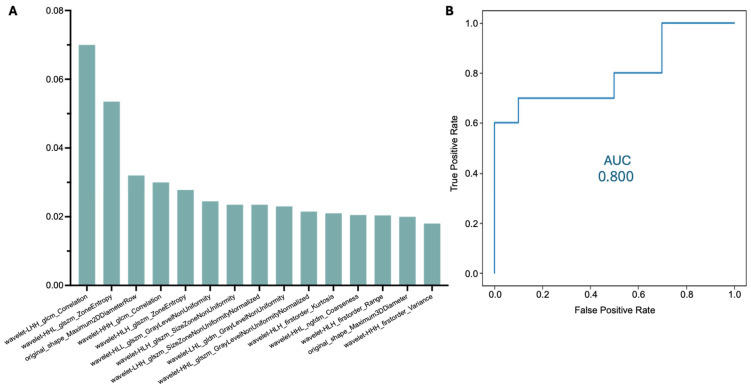
Classification of Top-15 radiomic features for disease prediction in the automatic dataset (**A**) and ROC curve showing the model’s performance in diagnosing myocardial pathology in the automatic segmentation dataset (**B**).

Radiomic features were extracted from a total of 100 CT scans. The radiomic feature extraction process led to a dataset of 843 extracted radiomic features per patient. Features with significant cross-variation between the two segmentation methods (manual vs. automatic) were eliminated, reducing the feature dataset to 591. A correlation analysis was carried out to address the issue of redundant features. Features with a correlation coefficient above 90% were systematically removed, further limiting the dataset to 139 radiomic features. These 139 features represented possible independent and significant predictors of myocardial pathology. This dataset was randomly divided into training and testing sets using an 80/20 split. Employment of a random forest classifier led to the identification of 15 key radiomic features that were highly predictive of myocardial disease. Following the implementation of random split training–testing and random forest classification, the overall model performance was evaluated. The model achieved an AUC score of 0.8 for predicting myocardial pathology using automatic annotations, supporting its ability to classify myocardial disease based on the extracted radiomic features (Figure 3).

**Figure 4:**
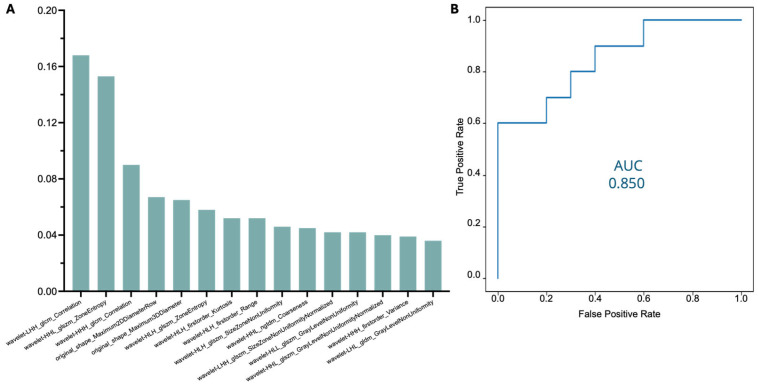
Classification of Top-15 radiomic features for disease prediction in the manual dataset (**A**) and ROC curve showing the model’s performance in diagnosing myocardial pathology in the manual segmentation dataset (**B**).

To further validate the robustness of these features, the 15 radiomic features identified from the automatic segmentation dataset were also analyzed within the manually annotated dataset. Upon applying the same random forest classifier, the model achieved an AUC score of 0.85 for disease prediction using manual segmentations (Figure 4).

**Figure 5:**
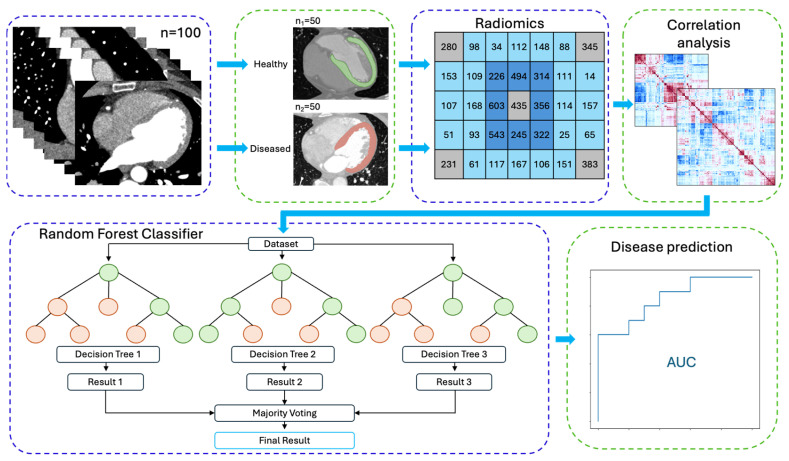
Illustration of automated disease classification model based on radiomic feature phenotyping.

This demonstrates that the selected radiomic features maintain their predictive accuracy, regardless of the segmentation process (manual vs. automatic). The consistency in performance across both segmentation approaches highlights the model’s robustness and its potential applicability in clinical settings for the automatic identification of myocardial disease. Figure 5 illustrates the disease classification model using radiomic feature extraction.

## 4. Discussion

The results of this study highlight the potential of ML and radiomic analysis in the field of cardiology, particularly for the detection and classification of myocardial diseases. The successful segmentation of LV myocardium from diverse CT scan protocols demonstrates the versatility and robustness of the ML model, which addresses key challenges in clinical imaging. Previous research efforts have demonstrated the feasibility of ML automatic LV segmentation using CT scans [10,11,13,14,15]. Such models are invaluable as they can reduce the time and expertise required to manually segment myocardial regions, which is particularly useful in resource-limited settings where highly trained image analysts may not be available. However, most studies rely on pristine CT datasets, typically incorporating exclusively CCTA scans. Such algorithms often fail to deliver accurate results when applied to other types of CT scans, resulting in poor generalizability.

In contrast, the automated LV segmentation algorithm developed in this study demonstrated strong performance in both the training and external validation phases (with a final Dice score of 0.907), even when tested on CT scans from different protocols. This score highlights the model’s ability to accurately replicate manual segmentations, which are typically regarded as the gold standard in cardiac imaging, regardless of the CT protocol used. The model’s ability to generalize across various CT protocols ensures its applicability across a wide range of clinical environments, allowing clinicians to incorporate this technology regardless of the specific imaging setup used.

Radiomic feature analysis has emerged as a highly promising tool in the study of cardiovascular diseases, with a particular focus on heart muscle disease. By extracting detailed imaging biomarkers that capture subtle tissue characteristics, radiomics has proven effective in enhancing the accuracy of diagnosis, improving prognosis, and enabling more precise monitoring of disease progression. Multiple studies have demonstrated the potential of radiomics to offer insights into myocardial pathology [16,17,18,19,20,21]. Most studies on heart muscle disease focus on CMR imaging, largely due to its superior ability to characterize tissue properties [19,20,21]. However, utilizing CT scans for texture analysis and disease classification can support large-scale opportunistic population screening by leveraging existing clinically acquired extensive datasets. The radiomic analysis in the present study led to the identification of 15 key radiomic features for disease prediction, further supporting the use of radiomics as a valuable biomarker for myocardial disease. The model showed a good ability to estimate the likelihood of underlying myocardial disease. The AUC of 0.8 for predicting myocardial disease in automatically derived LV segmentations demonstrates that these radiomic features offer significant diagnostic value, potentially aiding in the prompt automated screening and early detection of cardiomyopathies such as hypertrophic cardiomyopathy or ATTR-CM through CT scans. Early detection is particularly crucial in these conditions, as timely intervention can prevent the progression to heart failure and improve patient outcomes [22,23,24]. The results are comparable to those obtained using radiomic analysis based on manual LV segmentations (AUC = 0.85), further reinforcing the accuracy and consistency of the automated method.

From an interpretability standpoint, several of the top-ranked radiomic predictors in our model belong to feature families commonly associated with tissue heterogeneity and structural complexity (e.g., entropy, non-uniformity, and dispersion metrics), which have been leveraged in prior cardiac radiomics studies investigating myocardial scar/fibrosis and cardiomyopathy-related remodeling patterns [25,26]. Nevertheless, direct one-to-one pathological attribution of individual radiomic features remains an evolving area. Radiomic feature values can be influenced by acquisition and reconstruction parameters, scanner characteristics, and preprocessing choices, and the reproducibility of myocardial CT radiomics across technologies and protocols remains an important limitation [27]. Therefore, we intentionally avoided over-specifying mechanistic interpretations (e.g., attributing a specific feature uniquely to fibrosis or hypertrophy) and instead provide a cautious, feature-family-level biological context; future studies with larger cohorts and radiologic–pathologic correlation will be essential to validate disease-specific biological meaning and to strengthen clinical interpretability.

This study’s findings align with a growing body of research exploring the use of ML and radiomics for cardiovascular disease detection [24,28,29]. Prior studies have demonstrated the value of radiomic features in identifying myocardial infarction, fibrosis, and other pathological changes in cardiac tissues [30,31,32,33,34]. However, the present study differentiates itself by focusing on a wide range of myocardial diseases and demonstrating the model’s ability to generalize across multiple CT protocols. Moreover, while many studies have focused on radiomics analysis on manual or semi-automatic segmentations [7], this study validates a fully automated method of LV segmentation, radiomic feature extraction and disease prediction through radiomic analysis, highlighting the potential for AI to streamline clinical workflows, reduce the burden on human operators and aid in the prompt and non-invasive disease prediction [35]. By achieving an AUC of 0.8 for disease classification using automatic segmentations, the study underscores the feasibility of fully automated diagnostic systems for cardiomyopathies. This concept aligns with the broader paradigm of AI-enabled multi-organ phenotyping from clinically acquired CT scans. Recent studies on site-specific automated segmentation demonstrate how CT analysis can generate clinically relevant biomarkers without additional imaging [36,37].

Several challenges of this study should be acknowledged. One of the primary limitations of this study is the relatively small dataset used for training and validation. Although 60 scans were used for training and 10 for external validation, larger datasets are necessary to fully assess the model’s performance across different populations and imaging settings. A more extensive dataset, comprising patients from diverse demographic and clinical backgrounds, would help ensure the model’s generalizability and robustness. Additionally, the variability in CT scan quality and protocol, while addressed to some extent, remains a potential source of error. Although the model performed well across different CT protocols, future studies should explore the impact of further heterogeneity in imaging protocols, including the use of lower-resolution or artifact-prone scans.

Another important consideration is the reliability of the radiomic features extracted by the model. While this study rigorously reduced the number of features to the most stable and repeatable ones, the clinical utility of these features must be further validated. The features identified as critical for predicting myocardial disease may still vary between populations or disease subtypes, and their predictive value may be influenced by factors such as image acquisition settings or patient characteristics. Thus, while the study provides significant insights for the use of radiomics in myocardial disease classification, future work should focus on externally validating these features in larger, multi-center trials to ensure they remain robust and reproducible across different clinical contexts.

Lastly, the radiomic classification component relied on a single stratified 80/20 train-test split, and nested cross-validation or confidence interval estimation was not performed, which may limit the statistical robustness of the reported performance metrics; this design choice was made to preserve strict separation between training and testing data and to avoid overfitting given the limited sample size, in line with the proof-of-concept nature of the study. Calibration analysis was also not undertaken, as the present study focused on discrimination rather than probability calibration in a proof-of-concept setting. Clinical covariates were not incorporated into the predictive models, as the primary aim was to evaluate a fully imaging-driven, automated pipeline. Future studies with larger cohorts should integrate nested validation frameworks, calibration assessment, and combined imaging–clinical models to further strengthen clinical applicability.

## 5. Conclusions

This study developed and validated an ML model that effectively segments the LV myocardium from CT scans and uses radiomic feature phenotyping to predict the presence of underlying myocardial disease. The model achieved high segmentation accuracy with a Dice score of 0.907 and demonstrated robust predictive performance in automatically segmented scans for disease classification, with an AUC of 0.8. These findings underscore the potential of AI and radiomics to aid in the early detection as well as monitoring of heart muscle diseases.

## Data Availability

The original contributions presented in this study are included in the article/Supplementary Material. Further inquiries can be directed to the corresponding author.

## Supplementary Materials

The following supporting information can be downloaded at: https://www.mdpi.com/article/10.3390/jimaging12030120/s1, Table S1: Performance metrics of AI segmentation model after model training. Table S2: External validation of AI segmentation model. Performance metrics for each of the 10 CT scans used in the external validation of the AI model, along with the average performance metrics across all scans.

## Abbreviations

The following abbreviations are used in this manuscript:

AI	Artificial Intelligence
ATTR-CM	Transthyretin Amyloid Cardiomyopathy
AUC	Area Under the Curve
CMR	Cardiovascular Magnetic Resonance
CT	Computed Tomography
CTA	Computed Tomography Angiography
CCTA	Coronary Computed Tomography Angiography
ECG	Electrocardiogram
FPN	Feature Pyramid Network
HCM	Hypertrophic Cardiomyopathy
ICD	Implantable Cardioverter-Defibrillator
LV	Left Ventricle
ML	Machine Learning
ROC	Receiver Operating Characteristic
TTR	Transthyretin
