# Supplementary material for: Decoding the Heart Through Computed Tomography: Early Cardiomyopathy Detection Using Ensemble-Based Segmentation and Radiomics
Source: J Imaging. 2026 Mar 10;12(3):120. doi: 10.3390/jimaging12030120 (PMC13027443; doi:10.3390/jimaging12030120)
Supplement: Supplementary file 1 [file jimaging-12-00120-s001.zip › jimaging-4165907-supplementary.pdf]

Supplementary Material

Decoding the Heart Through Computed Tomography: Early Cardiomyopathy Detection Using Ensemble-Based Segmentation and Radiomics

FOLD	IOU	F1	F2	PRECISION	RECALL
1	0.799	0.888	0.911	0.852	0.928
2	0.828	0.906	0.914	0.893	0.92
3	0.8	0.888	0.892	0.883	0.895
4	0.772	0.87	0.875	0.869	0.879
5	0.783	0.878	0.891	0.858	0.9
6	0.762	0.864	0.873	0.851	0.88
7	0.778	0.869	0.867	0.878	0.866
8	0.761	0.86	0.853	0.873	0.848
9	0.802	0.889	0.903	0.868	0.913
10	0.828	0.906	0.901	0.915	0.899
**Average**	**0.791**	**0.882**	0.888	0.874	0.893

Table S1. Performance metrics of AI segmentation model after model training.

Patient	IOU	F1	F2	PRECISION	RECALL	SPECIFICITY
RMAI4HD_245_A	0.873	0.932	0.937	0.924	0.940	0.996
RMAI4HD_246_A	0.864	0.927	0.939	0.908	0.947	0.996
RMAI4HD_244_A	0.829	0.906	0.895	0.926	0.888	0.996
RMAI4HD_247_A	0.764	0.866	0.898	0.817	0.921	0.992
RMAI4HD_248_A	0.850	0.919	0.942	0.883	0.958	0.996
RMAI4HD_2410_A	0.838	0.912	0.911	0.912	0.911	0.997
RMAI4HD_242_A	0.864	0.927	0.925	0.930	0.924	0.998
RMAI4HD_243_A	0.848	0.917	0.919	0.914	0.921	0.997
RMAI4HD_249_A	0.866	0.928	0.915	0.951	0.907	0.998
RMAI4HD_241_A	0.725	0.840	0.876	0.787	0.902	0.997
**Average**	**0.832**	**0.907**	0.916	0.895	0.922	0.996

Table S2. External validation of AI segmentation model.
Performance metrics for each of the 10 CT scans used in the external validation of the AI model, along with the average performance metrics across all scans.
